# Solitary Abdominal Wall Lymph Node Recurrence in Prostate Cancer Patient with Dramatic Prostate-Specific Antigen Decrease following Metastasectomy

**DOI:** 10.1155/2023/7301284

**Published:** 2023-02-10

**Authors:** Andrew Williams, Amr A. Elbakry, Tyler Trump, Ion Prisneac, Matthew Smolkin, David Zekan, Mohamad W. Salkini

**Affiliations:** ^1^West Virginia University, School of Medicine, Morgantown, West Virginia, USA; ^2^Urology Department, West Virginia University Hospital, Morgantown, West Virginia, USA; ^3^Pathology Department, West Virginia University Hospital, Morgantown, West Virginia, USA

## Abstract

Prostate cancer patients routinely undergo surveillance for recurrence using prostate-specific antigen (PSA). While PSA's benefit in screening is controversial, its use for detecting recurrence in patients with history of prostate cancer is pivotal. Rising PSAs with the newly advanced prostate-specific membrane antigen positron emission tomography (PSMA PET) can help localize the location of recurrences for better excision and management. Here, we present a 55-year-old with prostate cancer, with initially undetectable postprostatectomy PSA levels, who later presented with a PSA of 3.47 ng/mL. PSMA PET showed isolated uptake in an abdominal wall mass. Pelvic lymphadenectomy and abdominal wall mass excision were performed, confirming a single metastasis in an abdominal wall lymph node. Metastasectomy led to a dramatic drop in PSA to 0.10 ng/mL both postoperatively and on long-term follow-up. Our case illustrates the potential benefit of metastasis-directed therapy in delayed oligometastasis following definitive management of prostate cancer.

## 1. Introduction

Prostate cancer is the second leading cause of death in American men. Currently, prostate-specific antigen (PSA) is the most common prostate cancer screening method used for patients between 55 and 69. While PSA's benefit in screening is controversial, its use for detecting recurrence in patients with history of prostate cancer is pivotal. Rising PSA in patients after prostatectomy is concerning for persistent or recurrent disease [1]. Recurrence universally occurs with an elevation in PSA [1]. A relatively new technique for detecting cancer metastasis is with prostate-specific membrane antigen positron emission tomography (PSMA PET) imaging which utilizes a radiotracer preferentially taken up by prostate tissue [2]. PSMA PET imaging helps detect sources of biochemical recurrence that were previously difficult to discern on computed tomography (CT), magnetic resonance imaging (MRI), or ultrasound. PSA elevation after definitive therapy is associated with increased risk of metastasis and death from prostate cancer [3]. Here, we present a patient with history of prostate cancer who was found to have biochemical recurrence 8 years after prostatectomy, and only a single lesion on PSMA PET imaging was detected.

## 2. Case Presentation

**Figure 1:**
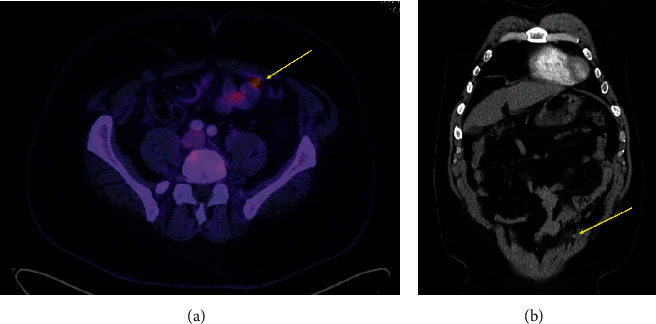
(a) Axial image of PSMA PET showing abdominal wall mass concerning for recurrence. (b) Coronal image of CT scan showing small abdominal wall mass correlating with the mass on PSMA PET scan.

**Figure 2:**
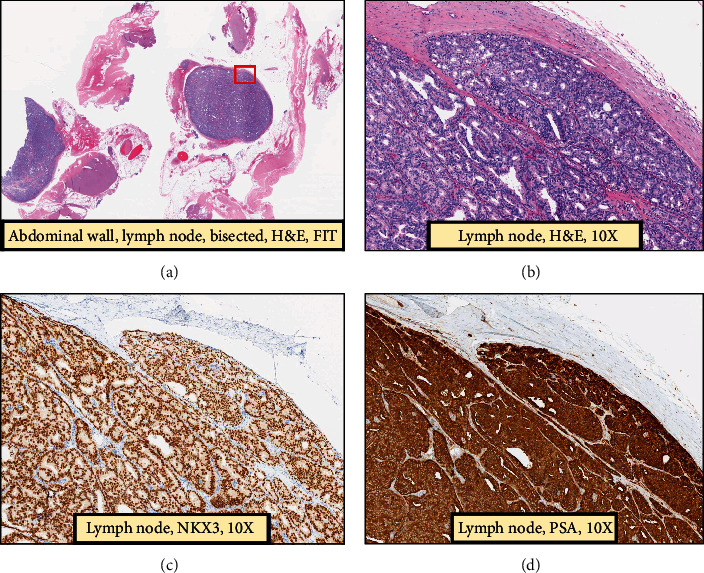
Microscopic examination of abdominal wall lymph node with H&E staining (a), with 10x magnification (b) showing that LN is extensively involved by neoplastic glands. Positive nuclear stain for NKX3 (c) and cytoplasmic stain for PSA (d) confirming prostate origin of the tumor.

A 55-year-old male initially was referred to urology clinic in 2014 because he had imaging after a trauma incidentally revealing a renal cyst. During his urology visit, he opted to have PSA checked which was found to be 10.3 ng/mL. He underwent prostate biopsy which revealed Gleason 4 + 3 prostate cancer with perineural invasion. He underwent robotic-assisted laparoscopic bilateral nerve-sparing radical prostatectomy. Pathology confirmed pT2cNx prostatic adenocarcinoma, with Gleason score 4 + 3, with perineural invasion, but no lymphovascular invasion, nor extracapsular extension. Apical margin was found to be positive. 38 fractions of adjuvant external beam radiation therapy (68.4 Gy at 1.8 Gy/fx) were provided postoperatively due to the positive margin. The patient recovered well from his treatment with little complications other than mild erectile dysfunction 3 years after surgery treated successfully with sildenafil. The patient was compliant with surveillance for 4 years after surgery, and his PSA remained <0.1 ng/mL until he was lost to follow-up for 3 years from 2018 to 2022. When he returned in 2022, his PSA was 2.3 ng/mL, indicating biochemical recurrence. Three months later, PSA increased up to 3.47 ng/mL. PSMA PET scan was obtained and revealed suspicious uptake in the left side of the abdominal wall within a 7 mm soft tissue nodule abutting the left rectus abdominis muscle (Figure 1). Other than the abdominal wall lesion, the patient had no other concerning uptake. The patient underwent robotic-assisted laparoscopic excision of the suspicious abdominal wall mass and pelvic lymph node dissection. Pathological examination revealed that abdominal wall mass was consistent of a group of abdominal wall lymph nodes. One abdominal wall lymph node (LN) was found to be positive for prostatic adenocarcinoma (Figures 1(a) and 1(b)). This was confirmed with positive nuclear stain for NKX3 and cytoplasmic stain for PSA (Figures 2(c) and 2(d)). Four other abdominal LNs were negative. The 19 pelvic LNs that were excised were found to be negative for malignancy, too. Of note, this site of recurrence was remote from prior port site placement. Surprisingly, his PSA became undetectable (≤0.1 ng/dL) after excision of the abdominal wall LN, confirming a single recurrent lesion 8 years after initial prostatectomy. Due to the positive LN, the patient has since been referred to medical oncology for androgen deprivation therapy (ADT) and docetaxel and continues to do well.

## 3. Discussion

The patients with localized prostate cancer are treated with the goal of curative therapy, whether through radical prostatectomy or radiation therapy with or without ADT [1]. The goal of prostate cancer treatment is to minimize the risk of recurrence, while preserving continence and sexual potency [1]. Regardless of treatment modality, follow-up should include regular monitoring of PSA levels to assess for biochemical recurrence. A rise in PSA is the first sign of recurrence, usually occurring earlier than clinical symptoms [3]. Recent advances in imaging modalities like PSMA PET for prostate cancer have led to highly accurate, reproducible, and safe method for detection of metastases, previously difficult to find on conventional imaging, particularly at low PSA levels [2]. PSMA PET imaging has a positive predictive value ranging from 84% to 92% for PSA ≥ 1.0 with false positives mainly occurring in the prostate or prostate bed [2].

When limited recurrences, often labeled oligometastases (≤3 sites of recurrence), are identified, the next step in management is somewhat debated. Management options include ADT, salvage lymph node dissection, or salvage radiation therapy. Those options can have a markedly variable success rates [4]. Metastasectomy has been reported in a few case reports to reduce PSA to undetectable levels, like our patient, suggesting a benefit for patient survival and cancer cure [5]. The surveillance or metastasis-directed therapy for oligometastatic prostate cancer recurrence (STOMP) trial hopes to characterise the benefit of metastasis-directed therapy (MTD) by randomizing patients to receive either active surveillance or MTD followed by active surveillance in patients with oligometastases. This may shed better light into the benefit of MTD therapy and metastasectomies in prostate cancer patients.

## 4. Conclusion

PSMA PET scan is a promising tool for accurate detection of recurrence or metastasis in patients with biochemical recurrence after definitive prostate cancer treatment. Metastasectomy in patients with oligometastases can lead to achieving undetectable PSA indicating a potential for survival benefit.
